# Construction of N-7 methylguanine-related mRNA prognostic model in uterine corpus endometrial carcinoma based on multi-omics data and immune-related analysis

**DOI:** 10.1038/s41598-022-22879-6

**Published:** 2022-11-05

**Authors:** Junde Zhao, Jiani Zou, Wenjian Jiao, Lidong Lin, Jiuling Wang, Zhiheng Lin

**Affiliations:** 1 Shandong University of Traditional Chinese Medicine, Jinan, 250014 Shandong China; 2 Office of Medical Insurance Management, Qilu Hospital of Shandong University, Jinan, 250012 China

**Keywords:** Computational biology and bioinformatics, Databases, Cancer, Gynaecological cancer, Tumour biomarkers

## Abstract

N-7 methylguanine (m7G) is one of the most common RNA base modifications in post-transcriptional regulation, which participates in multiple processes such as transcription, mRNA splicing and translation during the mRNA life cycle. However, its expression and prognostic value in uterine corpus endometrial carcinoma (UCEC) have not been systematically studied. In this paper, the data such as gene expression profiles, clinical data of UCEC patients, somatic mutations and copy number variants (CNVs) are obtained from the cancer genome atlas (TCGA) and UCSC Xena. By analyzing the expression differences of m7G-related mRNA in UCEC and plotting the correlation network maps, a risk score model composed of four m7G-related mRNAs (NSUN2, NUDT3, LARP1 and NCBP3) is constructed using least absolute shrinkage and selection operator (LASSO), univariate and multivariate Cox regression in order to identify prognosis and immune response. The correlation of clinical prognosis is analyzed between the m7G-related mRNA and UCEC via Kaplan–Meier method, receiver operating characteristic (ROC) curve, principal component analysis (PCA), t-SNE, decision curve analysis (DCA) curve and nomogram etc. It is concluded that the high risk is significantly correlated with (P < 0.001) the poorer overall survival (OS) in patients with UCEC. It is one of the independent risk factors affecting the OS. Differentially expressed genes are identified by R software in the high and low risk groups. The functional analysis and pathway enrichment analysis have been performed. Single sample gene set enrichment analysis (ssGSEA), immune checkpoints, m6A-related genes, tumor mutation burden (TMB), stem cell correlation, tumor immune dysfunction and rejection (TIDE) scores and drug sensitivity are also used to study the risk model. In addition, we have obtained 3 genotypes based on consensus clustering, which are significantly related to (P < 0.001) the OS and progression-free survival (PFS). The deconvolution algorithm (CIBERSORT) is applied to calculate the proportion of 22 tumor infiltrating immune cells (TIC) in UCEC patients and the estimation algorithm (ESTIMATE) is applied to work out the number of immune and matrix components. In summary, m7G-related mRNA may become a potential biomarker for UCEC prognosis, which may promote UCEC occurrence and development by regulating cell cycles and immune cell infiltration. It is expected to become a potential therapeutic target of UECE.

## Introduction

UCEC is one of the three major malignant tumors in gynecology. Its incidence ranks first among malignant tumors of the female reproductive tract in developed countries such as Europe and the United States^1^. The latest data show that the incidence and associated mortality of UCEC are on the rise worldwide with the delay in women’s reproductive age as well as the use of hormone drugs and the rejuvenation of comorbidities such as obesity, hypertension and hyperglycemia^2–4^. Although endometrial cancer is usually diagnosed early in the course of illness and generally has a good prognosis, a small number of patients present or develop into metastatic or recurrent UCEC, with a 5-year survival rate of only 10–20%^5,6^. The principal mode of initial treatment of UCEC is surgery. Corresponding adjunctive therapies are identified based on the recurrent risk and prognosis. Chemotherapy, targeted therapy, endocrine therapy and/or salvage radiotherapy are often used in the treatment of relapsed or advanced UCEC^7^. The overall prognosis is poor in view of recurrent or advanced UCEC. It has poor response to classical treatment. Therefore, it is urgent to explore new treatment approaches to improve prognosis.

m7G is one of the most common RNA base modifications in post-transcriptional regulation^8^. It is widely distributed in tRNA^9^, rRNA^10^ and the 5ʹ cap region of mRNA in eukaryotes^11^, playing a vital role in gene expression, processing, metabolism, protein synthesis and stable transcription^12^. The m7G modification is catalyzed by the Trm8/Trm82 complex in yeast and the METTL1/WDR4 complex in human beings^13^. This modification is closely related to tRNA stability and human diseases^14^. Recent studies have found that the m7G is also present inside the mRNA of higher eukaryotes. It may regulate almost every process of the mRNA life cycle, including transcription, mRNA splicing and translation, etc.^15–17^, suggesting that the presence of m7G modification sites in mRNAs may reveal new biological functions in future studies. At present, the research on identification of RNA modification sites is rapidly developing. However, the specific role and prognostic value of m7G-related mRNA remain unclear in the progression and metastasis of UCEC.

In this study, we aim to identify the m7G-related mRNAs with differential expression in UCEC, thereby constructing a new predictive prognostic risk score model and evaluating the model’s predictive performance in low-risk and high-risk patients. Secondly, we have established three clustering subtypes and systematically analyzed their relationships with prognosis, TME and immune infiltration. Overall, this study will provide new insights into the role of m7G-related mRNA in predicting UEC prognosis and immunotherapy efficacy.

## Materials and methods

### Data download and processing

The RNA-sequencing (RNA-seq) gene expression profiles and clinical information of 587 samples were downloaded from the TCGA database (https://portal.gdc.cancer.gov/), including mRNA expression data from 552 UEC tissues and 35 normal endometrial tissues. Then, 539 patients with sufficient gene expression profiles and OS data were selected for follow-up analysis based on patient clinical data and their corresponding RNA-seq data. The mutation data such as 529 cases of somatic mutations and CNV data were downloaded from UCSC Xena (https://xena.ucsc.edu/). The TCGA samples, as validation datasets were randomly divided into two equal numbers of groups and they were named Test1 and Test2, respectively. In addition, GSE119041 was downloaded from the Gene Expression Omnibus (GEO) database as an external cohort for validation. Gene expression data were annotated (http://asia.ensembl.org/homo_sapiens/info/index). The mean expression level of duplicated genes was calculated. All gene expressions were converted to log2 (gene expression value + 1) for follow-up analysis. A total of 29 RNA sequence datasets containing m7G modification sites were found in previous literature. The m7G gene expression level was obtained using the “LIMMA” package of R software and its expression profile was further normalized. The difference in m7G expression was compared using the Wilcoxon test between the normal sample group and tumor sample group.

### Differentially expressed gene screening, related functional annotation and pathway enrichment analysis

The TCGA-UCEC samples were divided based on the quartiles of m7G expression. The lowest 25% of quartiles were defined as the low m7G expression group and the highest 25% of quartiles were defined as the high m7G expression group under difference analysis. Differentially expressed m7G genes (DEGs) were screened out according to criteria of |log2 FC|> 0.1 and false discovery rate (FDR) < 0.05 in the UCEC low and high expression groups. Heat maps of DEGs were drawn by “pheatmap” R package. DEGs-related network diagrams were drawn based on the DEGs expression and the application of “igraph” and “reshape2” package. Entrez ID was converted through “org.Hs.eg.db” R package. Gene ontology (GO) enrichment and Kyoto Encyclopedia of Genes and Genomes (KEGG) signaling pathway analysis were performed on all DEGs using the “ClusterProfiler” R package.

### Analysis of gene mutations and copy number variants

Gene mutation maps of UCEC patients were drawn using “maftools” R package. A CNV matrix was established for 28 m7G-related CNV genes in UCEC. The CNV matrix is a binary matrix. GAIN represents the occurrence of the CNV for a particular gene in a particular sample. Other matrix elements include the LOSS. Visualizing graphs of CNV frequency were plotted using “barplot” and “RCircos” packages.

### Screening m7G-related prognostic mRNAs and constructing a prognostic risk model

The expression of 539 TCGA dataset samples was combined with clinical data to ensure that a prognostic model could predict effectively. Firstly, a univariate Cox proportional risk regression analysis of m7G-related DEGs was performed to obtain genes that were significantly related to prognosis (P < 0.05). Then, with survival status as the dependent variable and the selected gene expression value as the response variable, the number of genes was dimensionally reduced through Lasso COX regression analysis of 1000 times via glmnet software package, thus reducing the errors of the model to obtain a generalized linear model. After that, the multivariate Cox proportional risk regression analysis was performed to yield risk genes and construct a UCEC prognostic risk model. A disease risk score was used as a predictor of prognostic status in the model. The disease risk score was determined by the parameter X of the multivariate Cox proportional risk regression analysis and the expression Y of each gene in the sample. The risk score calculation formula is shown as follows:

$$\mathrm{Risk score }=\sum_{\mathrm{i}}^{\mathrm{n}}\mathrm{Xi}\times \mathrm{Yi}.$$

### Construction and efficacy evaluation of prognostic risk score model

The sample data were divided into high-risk and low-risk groups according to the median of risk indexes. Combined with survival information and gene expression, the survival curve was plotted to yield the survival conditions of patients with high and low risks and high and low gene expressions, respectively, as so to evaluate whether the predictive effect of the model was significant (P < 0.05). The statistical method used in this process was a log-rank test. The receiver operating characteristic curve (ROC curve) was plotted using “survival ROC” package of R software in order to evaluate the predictive power of regression models over 1, 3 and 5-year survival. When the AUC > 0.5 and it is closer to 1, the better the prognosis will be. Based on the gene expression in the prognostic model, the principal component analysis (PCA) and t-SNE analysis were performed using “prcomp” and “Rtsne” R packages, respectively. The results were visualized using “ggplot” R package. The univariate and multivariate Cox regression analysis was performed to evaluate the independent prognostic value of risk scores in this model. DCA curve analysis was carried out using the “stdca” software package of R language to evaluate the practical application of the model in the clinical practice. A nomogram in predicting the 1, 3 and 5-year survival rates of UCEC patients was produced by the “survival” package and the “rms” package of R software. The fit of the model was assessed by calibration diagram. The sensitivity of the model was evaluated by risk scores and ROC curves. The differentiation of the model was evaluated using risk scores and ROC curves.

### Functional enrichment analysis and immune-related analysis of differential genes in the two risk subgroups

Firstly, the “limma” R package was used to analyze the differential expression of genes between the high-risk and low-risk groups. Genes with |log2 FC|> 0.1 and FDR < 0.05 were differentially expressed between the high-risk and low-risk groups. The next step was to do the GO analysis. The infiltration scores of 16 immune cells and 13 immune functions were calculated for ssGSEA using the “GSVA” R package in UCEC. The activities of immune checkpoints and m6A RNA methylation regulatory factors were analyzed in the high-risk and low-risk groups. P < 0.05 was considered significant in all statistical analysis.

### GSEA analysis of m7G genes between high and low expression groups

In order to elucidate the significant differences of m7G genes in function and pathway between the high and low expression groups, the gene set enrichment analysis (GSEA) was performed based on the UEC expression matrix in TCGA using the “clusterProfiler” package of R software^18–20^. Only FDR was q < 0.05, the gene set was considered to be significantly enriched.

### Consensus clustering analysis of m7G-related mRNA responses based on UCEC

In order further investigate the role of m7G-related mRNA in UCEC, we used the “ConensusClusterPlus” package to divide those UCEC patients of the TCGA cohort into groups and obtain the optimal number of clusters based on the cumulative distribution function (CDF). The differences in genotyping were compared based on the expression level of prognostic risk genes. The Kaplan–Meier survival curve was used to analyze the differences between the OS and PFS among different groups. Based on the sample expression level of UCEC patients in the TCG database, the matrix scores and immunity scores were calculated using the “ESTIMATE” package among genotypes. In order to quantify and analyze the immune cell infiltration levels of each genotyping sample, the “CIBERSORT” computational R package was used to plot heat maps and pairwise difference plots.

### Calculation of tumor mutation burden and correlation analysis

The risk scores were calculated based on the risk score formula and the expression of genes in each genotyping sample. According to the median of risk indexes, the genotyping data were divided into high-risk and low-risk groups. The “maftools” R package was used to draw the gene mutation maps of patients in both risk subgroups. The difference in TMB was compared between the high-risk and low-risk groups. The correlation was compared between the TMB levels and risk scores. The new grouping is based on the Median value of TMB for the entire sample. Kaplan–Meier method was used to analyze the relationship of OS in the samples between the high and low TMB groups and the high-risk and low-risk groups within the TCGA cohort.

### Correlation analysis between risk scores and stem cells, TIDE scores and drug sensitivity

The “ggExtra” and “ggpubr” R packages were applied to calculate the stem cell correlation between the two risk subgroups and the “ggplot” package was applied to visualize the data results. The TIDE scores were compared between the high-risk and low-risk groups and a violin diagram was plotted. Finally, in order to further guide the clinical precision medications, two R packages of “Limma” and “pRRophetic” were used to analyze the data of the two risk subgroups the “ggboxplot” was introduced to visualize the drug sensitivity.

### Cell culture

Human endometrial cancer cell line HEC-1A (purchased from Shanghai Fuhengsheng Materials Co., LTD.) was cultured in Mccoy’s 5A + 10%FBS (both purchased from Gibco), and normal endometrial epithelial cells (purchased from Shanghai Fuhengsheng Materials Co., LTD.) were cultured in EMEM complete medium at 37 °C. 5%CO2, pH 7.2–7.4, sterile constant temperature culture. When the degree of cell fusion reached 90%, cell passage was carried out.

### Rt-PCR

Total RNA was collected using a RNeasy mini kit (RNA Fast 200, China), according to the manufacturer’s instructions. Then, cDNA was synthesized from 1 μg of total RNA using a SureScript First-Strand cDNA Synthesis Kit (GeneCopoeia, USA). Finally, the expression levels of NSUN2, NUDT3, LARP1, NCBP3. The sequences of the primers were as follows:

- NSUN2: forward 5′-ACCTGGCTCAAAGACCACACAG-3′, Reverse 5′-TGGCTTGATGGACGAGCAGGTA-3′.
- NUDT3: forward 5′-GAAGCACAGGACGTATGTCTATG-3′, reverse 5′-CTGCACGGGTTTGTGATACTG-3′.
- LARP1: forward 5′-GCTGTTTAGGAACAGCTGCC-3′, reverse 5′-CCACAGG TGACAGGGAGAAG-3′.
- NCBP3: forward 5′-AGGAAATCGGCGTCCAAGTT-3′, reverse 5′-TGCCTTGCCAGTCTTTGTCT-3′.
- GADPH: forward 5′-GCACCGTCAAGGCTGAGAAC-3′, reverse 5′-TGGTGAAGACGCCAGTGGA-3′.

Melting curve analysis was used to confirm the amplification specificity. The quantification data were analyzed using LightCycler analysis software (version 4.0; Roche Applied Science, Mannheim, Germany). Relative expression was normalized to that of GAPDH.

## Results

### Transcriptomic difference analysis in m7G low and high expression groups in patients with UCEC

**Figure 1:**
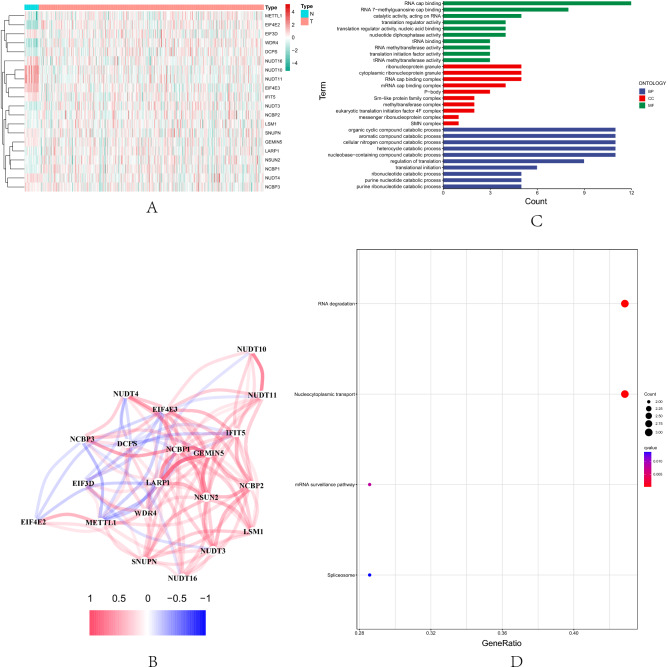
Transcriptomics analysis of m7G high and low expressions in UCEC tissues. (**A**) The heat map shows 20 DEGs. (**B**) Correlation network diagram of DEGs. (**C**) GO enrichment analysis results. (**D**) KEGG enrichment analysis results.

According to the m7G expression quartiles, TCGA-UCEC tumors were divided into two groups with high and low m7G expressions for differential expression analysis to identify 20 DEGs (|log2 FC|> 0.1, FDR < 0.05)with a difference of more than 2 times. The detailed distribution of 20 DEGs was shown by heat map (Fig. 1A) and a complete network of relationships among DEGs was plotted (Fig. 1B). The GO enrichment results showed that the DEGs of the m7G high and low expression groups were mainly involved in biological processes such as RNA cap binding, RNA 7-methylguanosine cap binding and catalytic activity, acting on RNA, etc. Its products were mainly involved in ribonucleoprotein granule, cytoplasmic ribonucleoprotein granule and RNA cap binding complex and other cellular components, playing a role in biological molecular functions, including organic cyclic compound catabolic process, aromatic compound catabolic process, cellular nitrogen compound catabolic process, heterocycle catabolic process and nucleobase-containing compound catabolic process, etc. (Fig. 1C). Meanwhile, the results of KEGG signaling pathway analysis showed that the m7G high expression was mainly enriched in RNA degradation, nucleocytoplasmic transport, mRNA surveillance pathway, spliceosome and so on signal pathways (Fig. 1D).

### Gene mutation map and CNV analysis of UCEC patients

**Figure 2:**
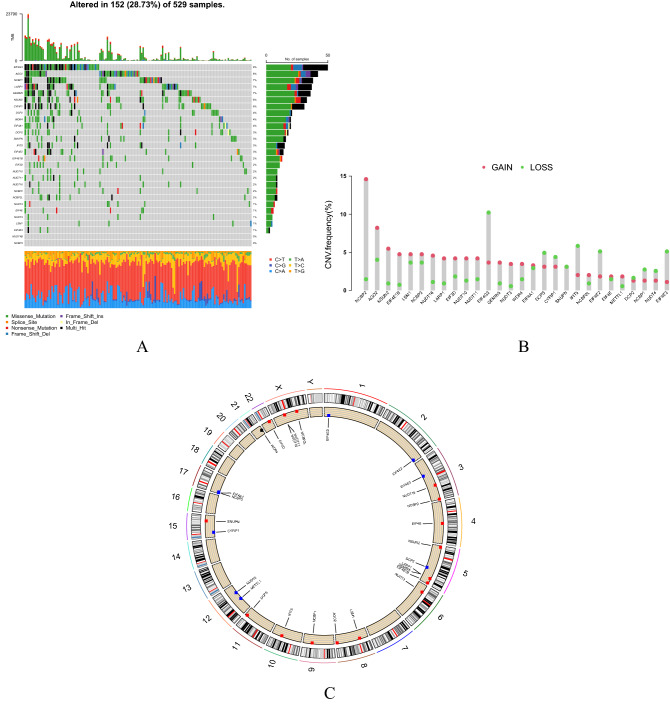
m7G-related gene mutations and CNV information in UCEC. (**A**) One hundred and fifty-two (28.73%) out of 529 patients show different genetic alterations. Of the missense mutation is the most common type of mutations. (**B**) 28 m7G-related gene copy number variants. (**C**) CNV change in location of intracellular m7G-related genes.

Of the 529 HCC patients in the TCGA database, 152 (28.73%) had genetic mutations (Fig. 2A). One of the most common types of gene mutations was the missense mutation. However, the proportion of missense mutations was small in some genes, such as EIF4G3 and LARP1. In addition, EIF4G3 mutations had the highest frequency, followed by AGO2, NCBP1, LARP1, GEMIN5, NSUN2, CYFIP1, DCP2, WDR4 and EIF4A1. However, NUDT4B and NCBP3 did not show any mutations. In order to analyze the copy number variants in the m7G-related genes, we selected 28 copy number variant genes in UCEC for further analysis. The status of m7G-related gene copy number variants in 28 UCEC patients was shown in Fig. 2B, where NCBP2, AGO2, NSUN2, EIF4E1B, LSM1, NCBP3 and NUDT16 had significant CNV amplification frequencies, and a small number of genes were missing, including EIF4G3, IFIT5, EIF4E2, and EIF4E3. Figure 2C showed the visual expression of all m7G-related copy number variant genes on chromosomes in the UCEC sample.

### Construction of prognostic model of m7G-related mRNA

**Figure 3:**
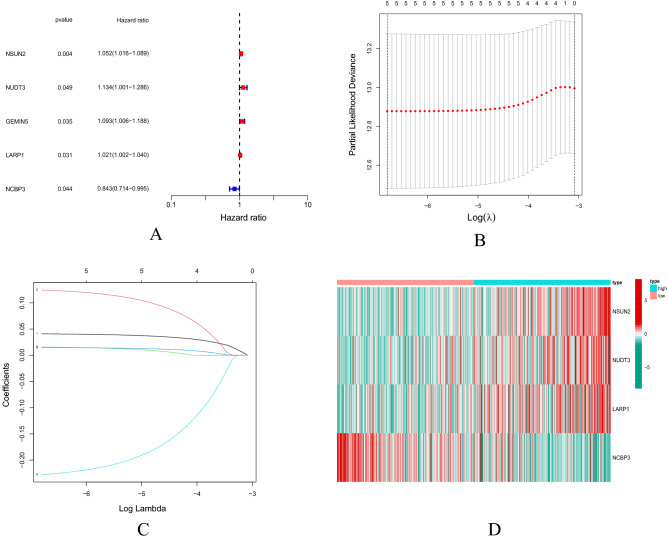
Construction of m7G-related mRNA prognostic model. (**A**) Screening prognostic mRNAs using univariate COX regression analysis. (**B**) LASSO regression analysis. (**C**) Multivariate COX proportional risk regression analysis of four prognostic mRNAs for establishment of a prognostic model. (**D**) Heatmap of prognostic-related m7G-related mRNAs. Green represents low expression of m7G-related mRNA, red represents high expression of m7G-related mRNA.

Firstly, in the TCGA cohort, the expression data of 20 m7G-related DEGs were extracted. The univariate COX regression was carried out for the overall survival (OS) to yield 5 m7G-related mRNAs significantly related to the OS (P < 0.05) (Fig. 3A). Then, 4 genes were obtained for subsequent analysis through Lasso COX regression analysis according to the optimal λ value (Fig. 3B,C). The multivariate COX proportional risk regression analysis was used to obtain a total of 4 risk genes (Fig. 3D), namely NSUN2, NUDT3, LARP1 and NCBP3 for the construction of a prognostic risk model.

### Construction of a risk score model based on m7G-related prognostic mRNA

**Figure 4:**
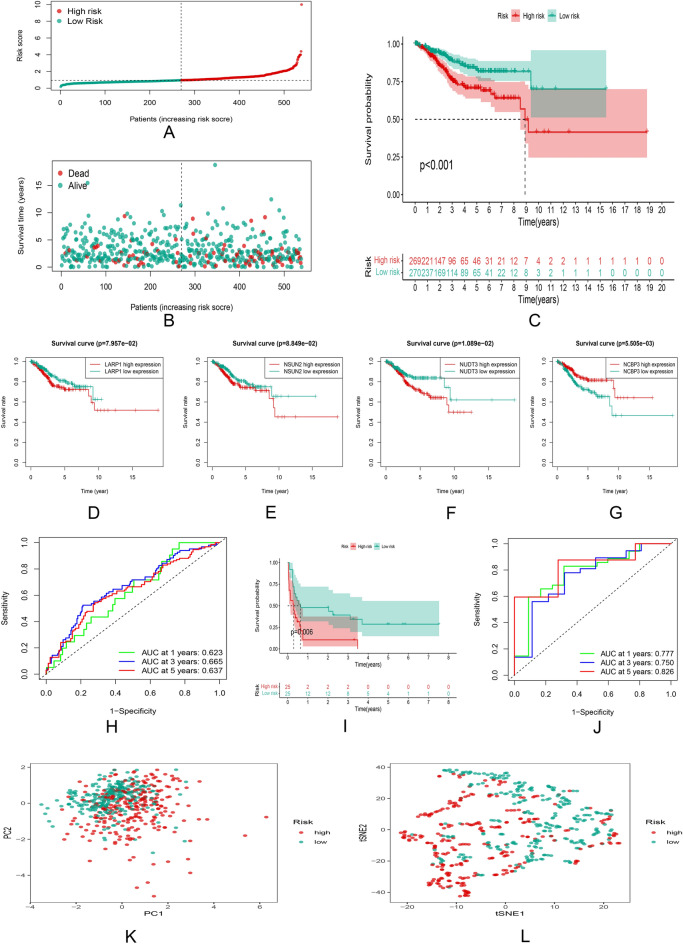
Survival analysis and comparison between the high and low risk groups. (**A,B**) Median distribution of risk scores and survival status of UCEC patients. (**C**) Kaplan–Meier survival curves of patients in the high and low risk groups in the TCGA cohort. (**D–G**) Survival status of high and low expression groups with NSUN2, NUDT3, LARP1 and NCBP3 genes. (**H**) ROC curves and AUC values of the model in the TCGA cohort. (**I**) PCA analysis results. (**J**) t-SNE analysis results.

The risk scores of all patients were calculated. All UCEC patients in the TCGA cohort were divided into high and low risk groups based on the median risk score. The distribution of risk scores and survival status was shown in Fig. 4A,B. The Kaplan–Meier survival curve showed that the patients with hepatocellular carcinoma in the high-risk group and those with NSUN2, NUDT3 and LARP1 genes in the high expression group had a lower survival rate and shorter survival time (Fig. 4C–F). The patients in the high expression group with the NCBP3 gene had a higher survival rate and longer survival times (Fig. 4G). In addition, the ROC curve showed that the model also had a good predictive power for overall survival of UCEC patients in the TCGA cohort, with AUC values of 0.623 (1 year), 0.665 (3 years) and 0.637 (5 years), respectively (Fig. 4H). At the same time, an external cohort and two sets of validation datasets were constructed to explain the generalization capacity of the models and the effect in other cohorts (Fig. 4I,J, Supplementary Figs. S1A, S2A). The patients in the low-risk group have a higher survival rate and longer survival times (Fig. 4I, Supplementary Figs. S1B, Fig S2B). The predictive power of the model was also validated in the external cohort (Fig. 4J). PCA and t-SNE analyses showed that the difference in sample data distribution was significant between the two risk subgroups in the TCGA cohort (Fig. 4K,L).

### Prognostic model as an independent prognostic factor in patients with UCEC

**Figure 5:**
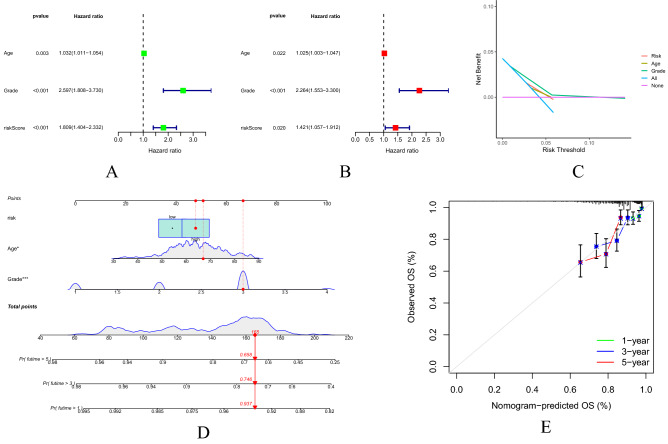
Independent prognostic analysis of risk score model. (**A,B**) Univariate and multivariate COX regression analysis of OS in the TCGA cohort. (**C**) DCA curve of scores and score-clinical variable integration mode. (**D**) Survival prognosis nomogram of UCEC patients. (**E**) Calibration charts for predicting 1, 3 and 5-year survival of UCEC patients.

Univariate and multivariate COX regression analysis was used to evaluate whether the prognostic model was an independent prognostic factor for the patients with UCEC. In the TCGA cohort, the univariate COX regression analysis showed a significant correlation [risk ratio (HR) 1.809; 95% confidence interval (CI) 1.404–2.332; P < 0.001] between the risk scores and overall survival of patients with UCEC (Fig. 5A). The multivariate COX regression further showed that the risk score was an independent prognostic factor for overall survival [HR 1.809; 95% CI 1.404–2.332; P = 0.020] (Fig. 5B). In order to understand the clinical effectiveness of the model, it was known from the DCA curves that the score-clinical variable integration model had a more significant net benefit than the traditional age, treatment protocols of all patients or non-treatment protocol in prognostic prediction (Fig. 5C). In the prognostic nomogram, each differential factor was based on the scores corresponding to the first ruler and the total score was obtained by added them up. Then according to the scores of the second ruler, the 1, 3 and 5-year survival rates were corresponded downward. As can be seen from the chart, the higher the age, the higher the score and the worse the survival prognosis (Fig. 5D). In clinical practice, a scoring system can be constructed for patients with different conditions, so as to obtain individualized survival predictions. In the calibration plot, the abscissa represented a predicted value and the ordinate represents an actual value. This curve and the 45° dashed line (i.e., an ideal state) represented the differences between the actual and predicted values. From the calibration chart, the 1-year survival prediction was close to the same with a high predictive value, while the 3 and 5-year survival prediction data were relatively close with some certain predictive value. Overall, the predictive model has a goodness of fit (Fig. 5E).

### Functional analysis in the TCGA cohort

**Figure 6:**
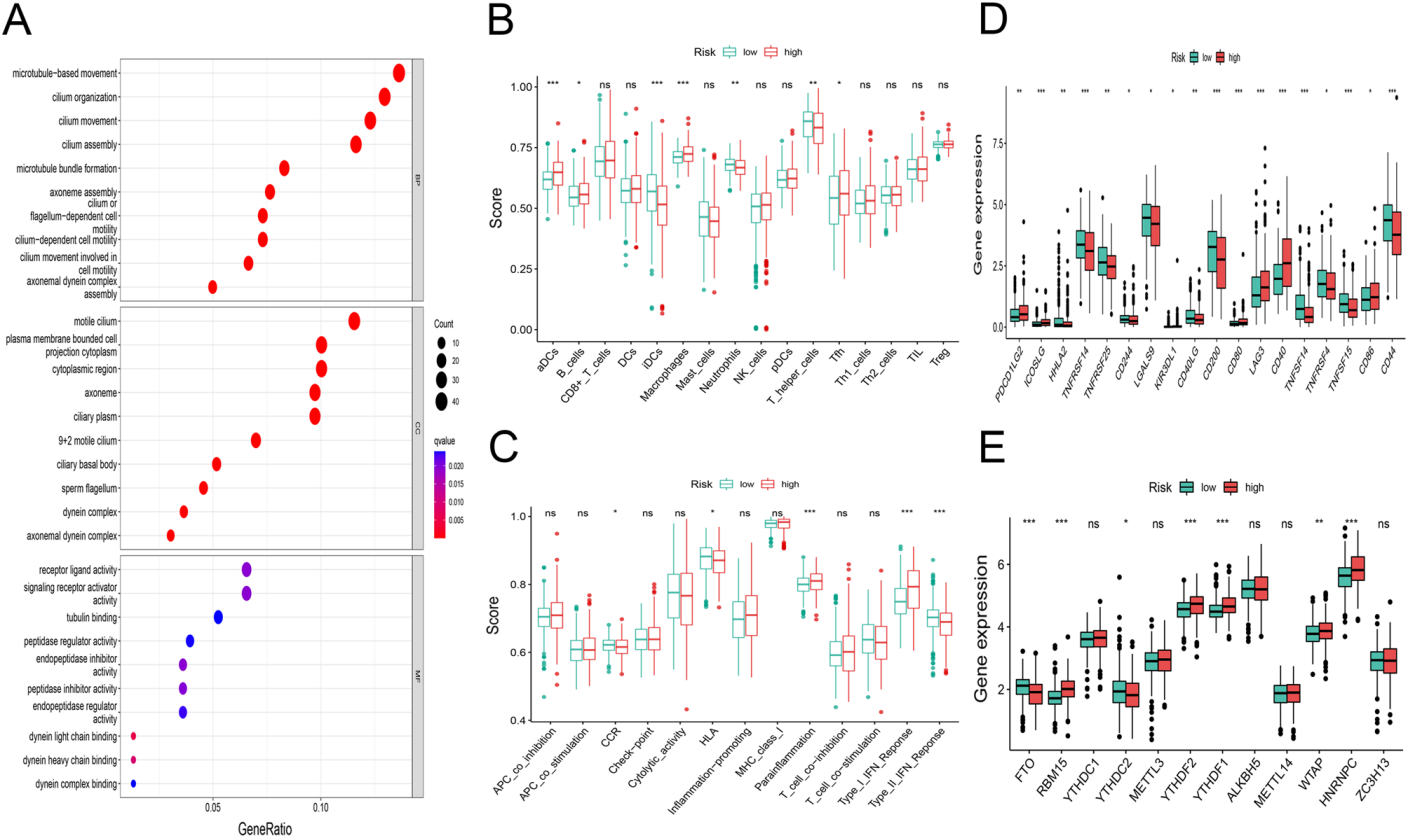
Functional enrichment analysis and immune infiltration levels of differential genes in the two risk subgroups. (**A**) GO analysis of differentially expressed genes. (**B**) Infiltration levels of immune cells. (**C**) Infiltration scores of immune pathways. Differential expression of 18 common immune checkpoints (**D**) and 12 M6A-related genes (**E**) between the two risk subgroups.

We screened out 205 differentially expressed genes [|log2 FC|> 0.1, FDR < 0.05] in the TCGA cohort. GO analysis showed that these differentially expressed genes were mainly enriched in the following aspects: microtubule-based movement, cilium organization, cilium movement, cilium assembly and so on biological processes (Fig. 6A). In order to further explore the relationship between the risk scores and the immunity, we used ssGSEA to quantify the infiltration degree of different immune cells and infiltration scores of immune pathways. The results showed the differences in infiltration of aDCs, B cells, iDCs, macrophages, neutrophils, T helper cells and Tfh were significant between the low-risk and high-risk groups in the TCGA cohort (Fig. 6B). The infiltration scores of immune functions such as CCR, HLA, Type II IFN Response were higher in the low-risk group than those in the high-risk group. Moreover, the difference was statistically significant (P < 0.05) between the two risk subgroups (Fig. 6C). In terms of immune checkpoints, the difference in immune-related genes was significantly between the two risk subgroups (P < 0.05). Moreover, the expression levels of HHLA2, TNFRSF14, TNFRSF25, CD244, LGALS9, CD40LG, CD200, TNFSF14, TNFRSF4, TNFSF15 and CD44 in the low-risk subgroup were higher than those in the high-risk subgroup (Fig. 6D). As shown in Fig. 6E, the expression levels of M6A-related genes RBM15, YTHDF2, YTHDF1, WTAP and HNRNPC in the high-risk subgroup were significantly higher than those in the low-risk subgroup (P < 0.01).

### GSEA-based m7G-related signal pathways

**Figure 7:**
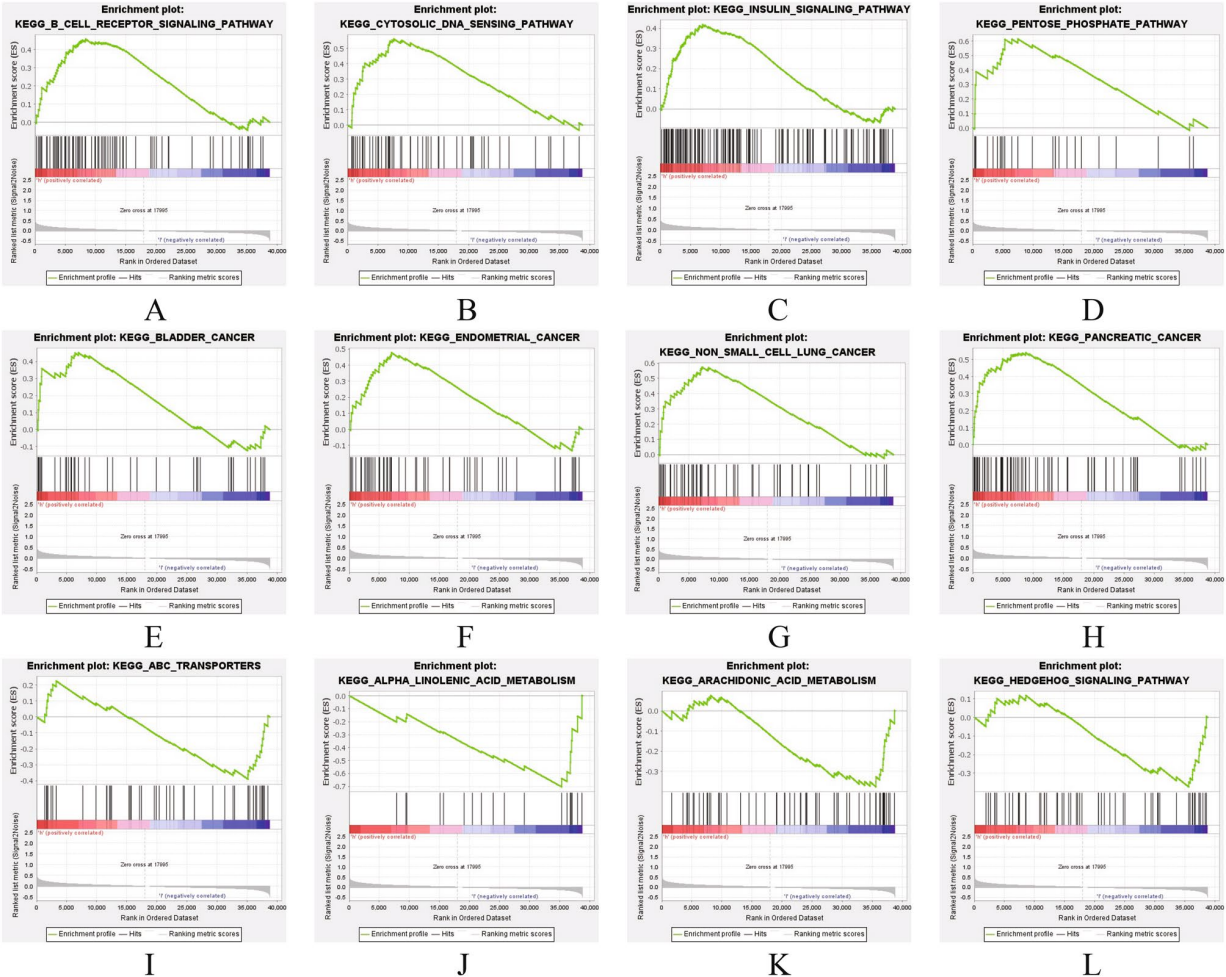
GSEA of M7G high expression (**A–H**) and low expression (**I–L**) samples.

The low and high expressions of m7G were detected with the “cluserPorfiler” package. The signal pathways of UECC participating in TCGAs were identified by GSEA analysis. Signaling pathways such as B CELL RECEPTOR, CYTOSOLIC DNA, PENTOSE PHOSPHATE and INSULIN were enriched in the m7G highly expressed UCEC phenotype (Fig. 7A–D), as well as significantly enriched in endometrial cancer, bladder cancer, non-small cell lung cancer and pancreatic cancer and other diseases (Fig. 7E–H). Biological processes such as alpha linolenic acid metabolism, transporters, hedgehog signaling pathway and arachidonic acid metabolism were enriched in the m7G lowly expressed phenotype (Fig. 7I–L).

### Analysis of survival, tumor microenvironment and immune cell infiltration among 3 genotypes

**Figure 8:**
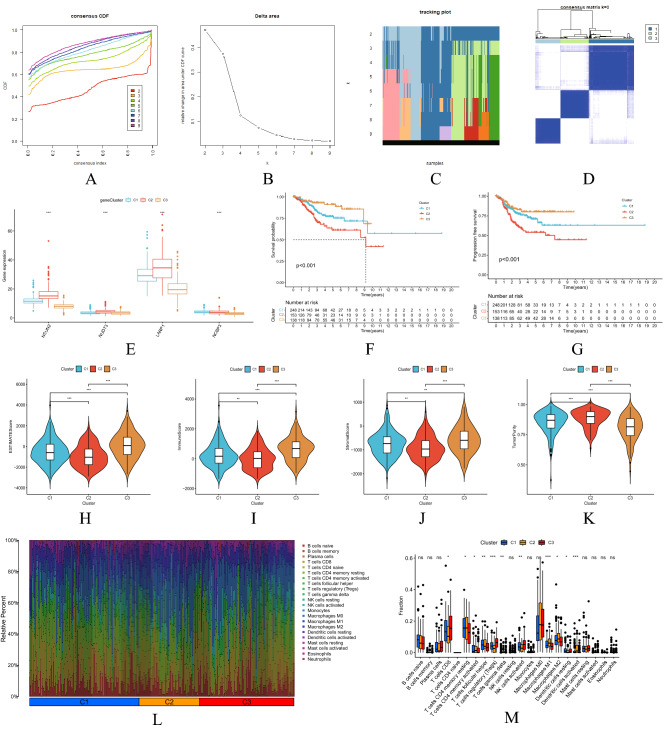
Survival, TME and immune cell infiltration analysis of consensus clustering recognition and typing. (**A**) Consensus clustering CDFs of k = 2–9. (**B**) Relative change in area under CDF curve of k = 2–9. (**C**) Tracking plot of k = 2–9. (**D**) Consensus clustering matrix of k = 3. (**E**) Differential distribution of 4 prognostic risk genes in 3 types among the three groups with respect to OS (**F**) and PFS (**G**) Kaplan–Meier survival curve. (**H**) ESTIMATEScore. (**I**) ImmuneScore; (**J**) StromalScore. (**K**) TumorPurity; Principal component analysis plot of immune cell infiltration matrix of 3 types (**L**) and variance analysis plot (**M**).

Based on the expression similarity of m7G-related mRNAs in the UCEC samples, the gene set was finally divided into three stable clusters by choosing k = 3, with the stability of clusters ascending between k = 2 and k = 9 (Fig. 8A–D). The differential distribution of the four prognostic risk genes in the three types was shown in Fig. 8E. As shown in the Kaplan–Meier survival curves in Fig. 8F,G, the difference in survival state was significantly among three groups. Besides, the OS and PFS of the third group were the best (P < 0.001). TME, ESTIMATE Score, Immune Score, Stromal Score, Tumor Purity were significantly correlated with (P < 0.01) types C1–3 (Fig. 8H–K).

Based on the gene expression profiles of these samples, CIBERSORT was used to analyze the main components of the immune cell infiltration matrix of three types. Figure 8L showed that the difference in immune cell infiltration was significant among the three types. After performing the differential analysis of the same immune cells for the 3 types, the results showed that the difference in T cells CD8, T cells CD4 memory resting, T cells CD4 memory activated, T cells follicular helper, T cells regulatory (Tregs), T cells gamma delta, NK cells activated, macrophages M1, macrophages M2, dendritic cells resting and dendritic cells activated was significant (all P < 0.05) among three types. See Fig. 8M.

### Significant correlation between the TMB levels and OS in patients with UCEC

**Figure 9:**
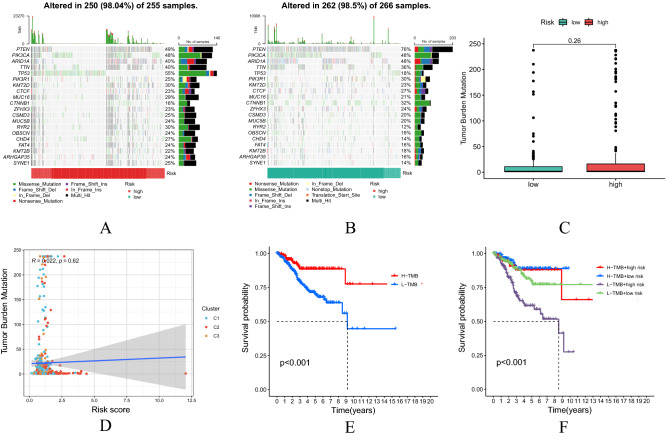
Correlation analysis of TMB levels between the two risk subgroups. (**A,B**) Distribution of the first 20 mutant genes in the high and low risk groups. (**C**) Difference analysis of TMB in the two risk subgroups. (**D**) Correlation between TMB and risk score. (**E,F**) Analysis of OS in UCEC patients in combination with TMB levels and risk scores.

Of the 255 UCEC patients in the high-risk group, 250 (98.04%) had genetic mutations in the gene mutation map (Fig. 9A) while 266 UCEC patients in the low-risk group, 262 (98.5%) had genetic mutations in the gene mutation map (Fig. 9B). There was no significant difference in TMB levels in the high-risk and low-risk groups (Fig. 9C). There was no significant correlation between the TMB levels and risk scores in UCEC patient samples (Fig. 9D). The TMB level in UCEC patients was calculated. Having combining risk scores with TMB levels, it was concluded that the patients with UCEC had a higher TMB level and longer survival time in the low-risk group (P < 0.001). See Fig. 9E,F.

### Risk score model closely related to stem cells, TIDE scores and drug sensitivity

**Figure 10:**
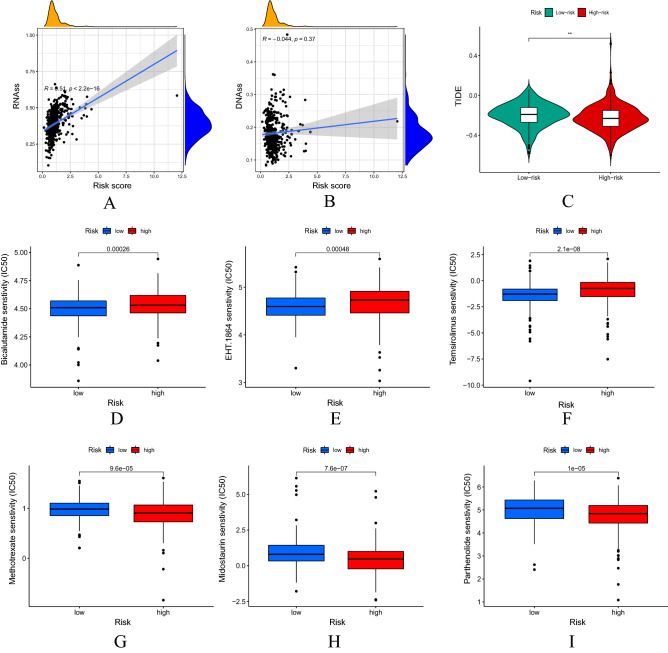
The risk score model is closely related to stem cells, TIDE scores and drug sensitivity. Correlation between the risk scores with RNAss (**A**) and DNAss (**B**) stem cells. (**C**) Differential expression of TIDE scores between the low and the high risk groups. (**D–I**) Drug sensitivity analysis of two risk subgroups.

As shown in Fig. 10A,B, Spearman correlation test suggested that the risk score model was positively correlated with RNAss (P < 0.001) and not significantly correlated with DNAss (P = 0.37). UCEC sample data in the low-risk group had scored higher in the TIDE scores and the difference was statistically significant (P < 0.01) between the two risk subgroups (Fig. 10C). Drug sensitivity analysis revealed that there was a difference in drug sensitivity (all P < 0.001) between the high-risk and low-risk groups. Moreover, the bicalutamide sensitivity (IC50) of bicalutamide, EHT.1864 and temsirolimus in the low-risk group was lower than that in the high-risk group (Fig. 10D–F). The IC50 of methotrexate, midostaurin and parthenolide in the high-risk group was lower than that in the low-risk group (Fig. 10G,I).

### RT-PCR

**Figure 11:**
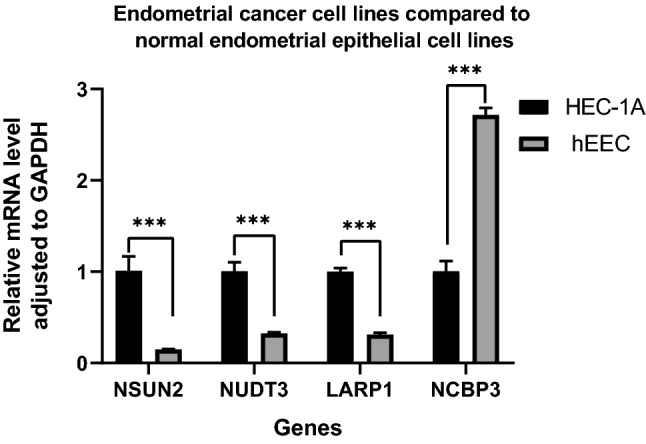
The result of RT-PCR.

Compared with normal endometrial epithelial cell line hEEC, the expression levels of NSUN2, NUDT3 and LARP1 in human endometrial carcinoma cell line HEC-1A were significantly increased (P < 0.001), while the expression levels of NCBP3 were significantly decreased (P < 0.001), further supporting the conclusion of the above analysis (Fig. 11).

## Discussion

UCEC is a common gynecologic malignant tumor. The principal mode of initial treatment for UCEC is surgery and corresponding adjuvant therapies decided based on the recurrent risk and prognosis. For the treatment of recurrent or advanced endometrial cancer, the targeted therapy, endocrine therapy, and/or palliative radiation therapy (radiotherapy) are mostly performed based on factors such as histological type, differentiation, lymph node metastasis and depth of muscle invasion (chemotherapy) etc. Non-focal recurrent or metastatic UCEC has a poor prognosis, with a 5-year survival rate of only 10–20%. Considering that there is currently no standard post-line treatment protocol for relapsed or advanced UCEC, poor overall prognosis and poor response to classical therapy, new prognostic targets and treatment strategies need to be explored to improve prognosis.

As one of the basic processes of epigenetic regulation, RNA modification is involved in many biological physiological and pathological functions^21–24^. Studies have revealed that RNA modification disorders promote the processing and translation of carcinogenic transcription subsets, and are closely associated with the metastasis and progression of prostate cancer, esophageal squamous cell carcinoma, gastrointestinal tumors and so on human cancers^25–29^. However, current research on the role of m7G-related mRNAs in the occurrence and development of UCEC is still limited. In this paper, we have constructed a risk score model for the first time based on m7G-related mRNA and systematically analyzed the prognostic and immunotherapeutic value of this model for patients with UCEC. Firstly, we have screened m7G-related mRNAs that are differentially expressed in UECC tissues and paracancer tissues and analyzed the correlation among the mRNAs. By combining the expression of UCEC samples in the TCGA dataset with clinical data, and performing univariate, Lasso COX regression analysis and multivariate Cox proportional risk regression analysis, risk genes are obtained and a UCEC risk prognosis model is established.

We have then comprehensively investigated the relationship between the four m7G-related mRNAs (NSUN2, NUDT3, LARP1 and NCBP3) and UCEC clinical data variables. Existing studies have shown that these genes play vital roles in the pathogenesis and progression of human cancer. AS a post-transcriptional regulatory factor, NOP2/Sun domain family member 2 protein (NSUN2) plays a key role in catalyzing tRNA methylation, promoting gene and protein translation and maintaining transcriptional stability^30–33^. Besides, NSUN2 has a dual role in tumor cells, playing different functions at different stages of the cell cycle in animal tissues. Although a study report reveals that the NSUN2 is overexpressed in epithelial tumor cells^34^ and ovarian cancer^35^ compared to normal tissue. Moreover, the patients with a low level of NSUN2 show a better overall survival rate than those with a high level of the NSUN2. However, in human skin cancer, the metastatic capacity of cancer cells is negatively correlated with the expression of the NSUN2^34^. In addition, some studies have shown that the NSUN2 has the properties of delaying protein synthesis and controlling the stem cell cycle in stem cells, which may lead to tumor recurrence and chemotherapy resistance^34^. NUDT3 is a cytoplasmic protein with mRNA dissociation activity in the family of diphosphoinositol polyphosphate phosphohydrolases (DIPP)^36^. It is found that through genome-wide and NUDT3 gene knockout cell analysis of NUDT3 damaged cells, NUDT3 is a mRNA dissociation enzyme that directly or indirectly affects the stability of mRNAs and regulates expression levels to adjust the cell migration, such as MCF-7 breast cancer cell migration^37^. In addition, NUDT3 has been found to be a key link in proliferative biological pathways. Small molecule drugs act as targeted agents for Triple-negative breast cancers (TNBCs) by inhibiting NUDT3^38^. La-related protein 1 (LARP1) belongs to RNA-binding proteins (RBPs). As one of the post-transcriptional regulatory factors, the carcinogenic properties of LARP1 has been demonstrated in many studies. LARP1 promotes the occurrence of NSCLC and CC by targeting its enhancers to positively regulate the expression of mTOR in non-small cell lung cancer (NSCLC) and cervical cancer (CC)^39,40^. At the same time, LARP1 can be used as a prognostic marker of colorectal cancer (CRC). Its overexpression is significantly correlated with the poor prognosis of the CRC and the OS^41,42^. In addition, LARP1 can also indirectly regulate the expression of bcl2 and BIK and the development of ovarian cancer^39^. Nuclear cap‐binding protein 3 (NCBP3) (or C17orf85) is a new cap-binding protein that may bind directly to RNA caps^43,44^. NCBP3 may act as a bridge between RBPs in the biological function of mRNA, upregulating the expression of downstream target genes in non-small cell lung cancer by interacting with NCBP1, thereby promoting the progression of lung cancer^45^. Furthermore, NCBP3 is found in glioma tissues and cells to bind directly to small nucleolar RNA host gene 6 (SNHG6) and inhibit the transcription of gastrulation brain homeobox 2 (GBX2) in a manner that relies on polycomb repressive complex 2 (PRC2), thereby promoting malignant progression of gliomas^46^. These results show that the four m7G-related mRNAs of NSUN2, NUDT3, LARP1 and NCBP3 are closely related to tumorigenesis and prognosis. This confirms our conclusions in UCEC.

It has been found that the UCEC could evade the immune system to achieve tumor progression and metastasis by mimicking the immune tolerance mechanisms that occur in the maternal fetus^47,48^. Currently, an immunotherapy regimen with immune checkpoint inhibitors—lenvatinib plus pembrolizumab has become an emerging field of progressive advanced UCEC research and treatment after systemic treatment^49–51^. We have divided the UCEC patients into high-risk and low-risk groups based on a risk score model and compared differentially expressed genes between the two risk subgroups. The results show that these four m7G-related prognostic mRNAs of NSUN2, NUDT3, LARP1 and NCBP3 in the risk score model are not only associated with the patient’s OS, but also significantly related to immune cell infiltration, immune checkpoints and immune-related signaling pathways, etc. These findings may contribute to further insight into the effects of m7G-related prognostic mRNA on UCEC prognosis and immunotherapy. A variety of prognostic markers have been proved to have considerable prospects in the immune and tumor microenvironment, providing a new direction for immunotherapy^52,53^. ssGSEA analysis shows a significant increase in immune cells that promote the tumor proliferation and metastasis such as infiltrating macrophages in the UEC samples of high-risk group^54,55^. The poor prognosis of patients in the high-risk group may be related to pro-cancer infiltrating immune cells. In addition, compared with the m7G-related prognostic mRNA low-risk group, UCEC samples in the high-risk group mostly have a lower level of immune checkpoint molecular expression, suggesting that the patients in the low-risk group are more likely to benefit from the treatment with immune checkpoint inhibitors. It also shows that our m7G-related prognostic mRNA risk score model may be able to predict the clinical efficacy of immune checkpoint blocking therapy in patients with UCEC.

In this paper, based on the expression similarity of m7G-related mRNAs in the UCEC samples, the gene set is finally divided into three stable clusters by choosing k = 3. There have been many previous reports on UCEC subtypes, such as the quantification of immunophenoscore (IPS) by by the pattern of gene differential expression and the construction of five immune molecular subtypes^56^. There are also methods such as clustering EC samples in TCGA into immune signature cluster modeling using GVSA enrichment analysis, evaluated immune cell profiling in UCEC cohorts and defined four immune subtypes of EC^57^.

Expression of TMB has gradually emerged as the best biomarker chosen for a variety of tumor immune checkpoints, including lung cancer, colorectal cancer, prostatic cancer and breast cancer^58^. In this study, we have obtained 3 genotypes based on consensus clustering analysis of UCEC responses to m7G-related mRNAs. Through somatic mutation analysis of UCEC samples in the TCGA database, we have found that the patients in the low-risk group have a higher level of TMB, and those with high TMB in the low-risk group have the best prognosis. As a novel biomarker, TMB has been associated with the efficacy of immunosuppressants on non-small cell lung cancer and malignant melanoma^59^. Some studies have suggested that the patients with a high TMB have a better prognosis in non-small cell lung cancer and malignant melanoma, compared with renal clear cell carcinoma, colon cancer and prostatic cancer^60^. This means that the patients with a low risk and a high TMB are likely to be recognized by immune cells and may benefit more from immunotherapy.

Bicalutamide, a first-generation non-steroidal androgen receptor (AR) antagonist, has become a key part of prostate cancer (PCa) treatment by acting directly on the AR^61^. In Hepatocellular carcinoma (HCC), low expression of SNHG1/SNHG3 was more sensitive to bicalutamide^62^. In a lncRNA prognostic risk model of gastric cancer patients, high-risk patients were more sensitive to chemotherapeutic agents such as bicalutamide compared to the low-risk group^63^.

### Limitations

However, our current research still has some shortcomings. Firstly, the AUC value of the verification group is not ideal, so more external databases and clinical samples are needed to validate its predictive ability. In addition, the specific mechanisms by which the 4 risk genes affect UCEC prognosis still need to be further studied, which may become a new target for the UCEC treatment.

## Conclusion

To sum up, in this study, bioinformatics methods have been used to analyze multi-omics data of UCEC samples in the TCGA database. The first UCEC prognostic risk score model is established based on 4 m7G-related mRNAs (NSUN2, NUDT3, LARP1 and NCBP3), which can make individualized predictions of survival rate in UCEC patients and is an independent prognostic factor for UCEC patients. In addition, this risk model has a certain reference value for predicting immune cell infiltration, immune function, immune checkpoints, TMB levels, m6A-related genes, tumor immune microenvironment, stem cell correlation, TIDE scores and drug sensitivity in UCEC, indicating that targeting m7G-related mRNA may become a promising target for the treatment of UCEC. It will provide new directions and ideas for follow-up clinical diagnosis and treatment of UCEC, and has great clinical significance for UCEC-specific therapeutic drugs.

## Supplementary Information

Supplementary Information 1.

Supplementary Information 2.

Supplementary Legends.

Supplementary Figure S1.

Supplementary Figure S1.

Supplementary Figure S2.

Supplementary Figure S2.

Supplementary Information 8.

## Data Availability

The datasets generated and/or analyzed during the current study are available in the [The Cancer Genome Atlas (TCGA)] repository, [https://portal.gdc.cancer.gov/] and UCSC Xena repository [https://xenabrowser.net/datapages/].
