# Supplementary material for: Construction of N-7 methylguanine-related mRNA prognostic model in uterine corpus endometrial carcinoma based on multi-omics data and immune-related analysis
Source: Sci Rep. 2022 Nov 5;12:18813. doi: 10.1038/s41598-022-22879-6 (PMC9637130; doi:10.1038/s41598-022-22879-6)
Supplement: Supplementary file 3 — Supplementary Legends. [file 41598_2022_22879_MOESM3_ESM.docx]

**Supplementary Figure Legend:**

**Supplementary Figure S1A-B:** Survival curves and ROC curves for the Test1 group.

**Supplementary Figure S2A-B:** Survival curves and ROC curves for the Test2 group.
