# Supplementary figures and images for: Construction of N-7 methylguanine-related mRNA prognostic model in uterine corpus endometrial carcinoma based on multi-omics data and immune-related analysis
Source: Sci Rep. 2022 Nov 5;12:18813. doi: 10.1038/s41598-022-22879-6 (PMC9637130; doi:10.1038/s41598-022-22879-6)

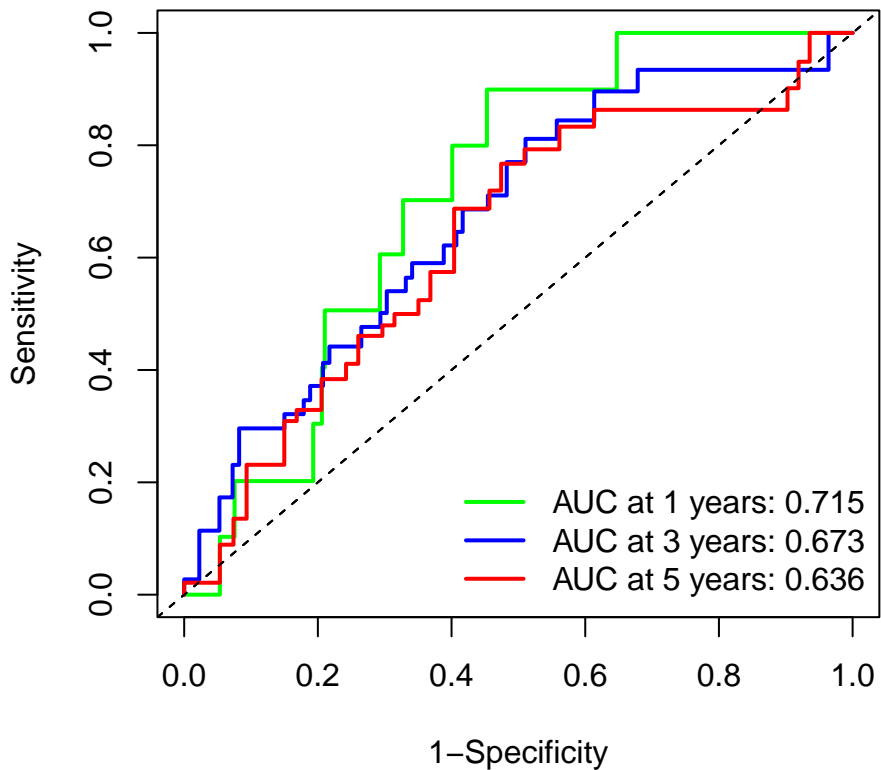

Supplement: Supplementary file 4 — Supplementary Figure S1. [file 41598_2022_22879_MOESM4_ESM.pdf]

Survival probability

Risk High risk Low risk

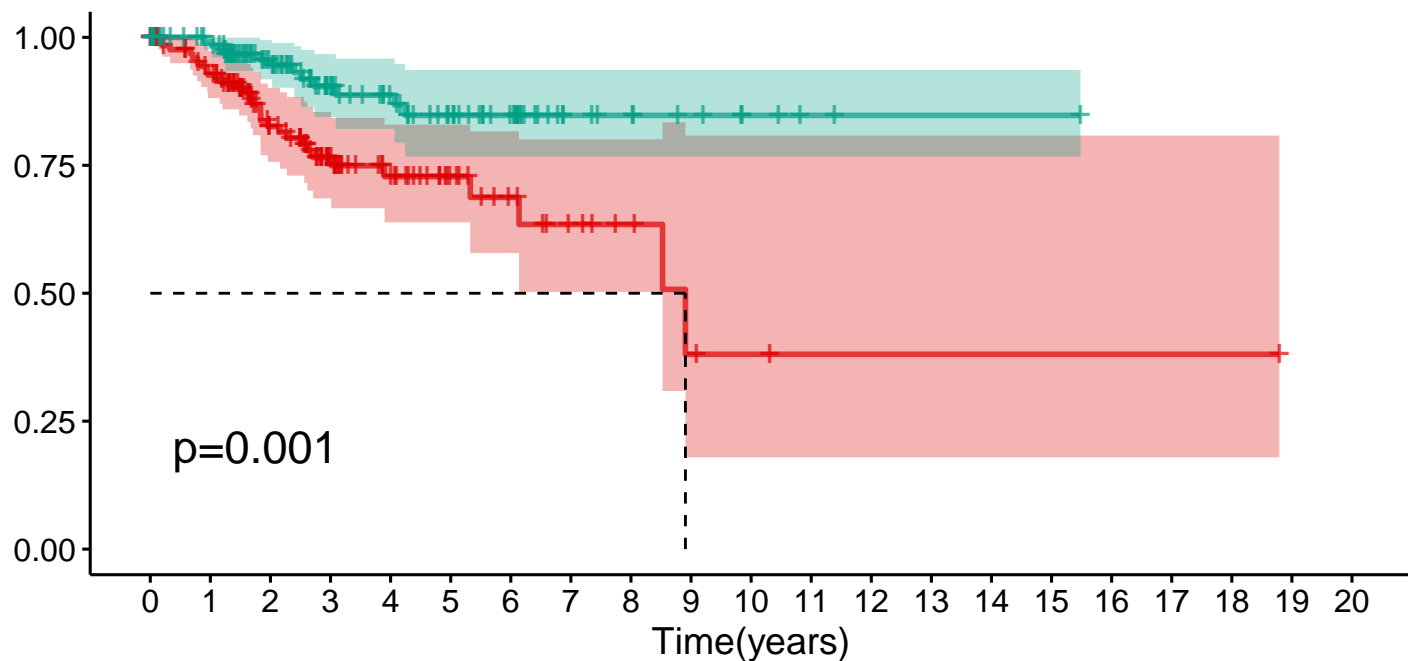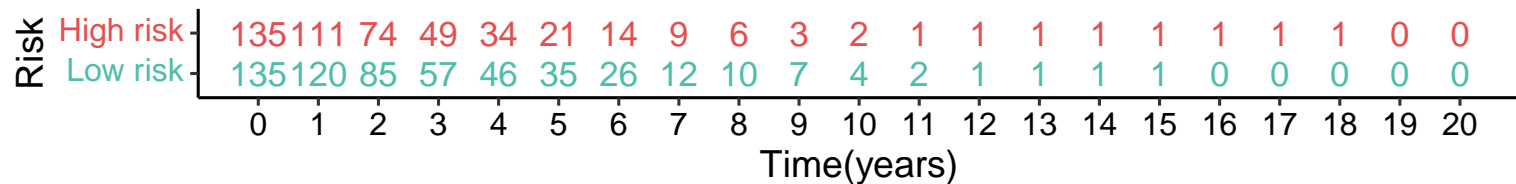

Supplement: Supplementary file 5 — Supplementary Figure S1. [file 41598_2022_22879_MOESM5_ESM.pdf]

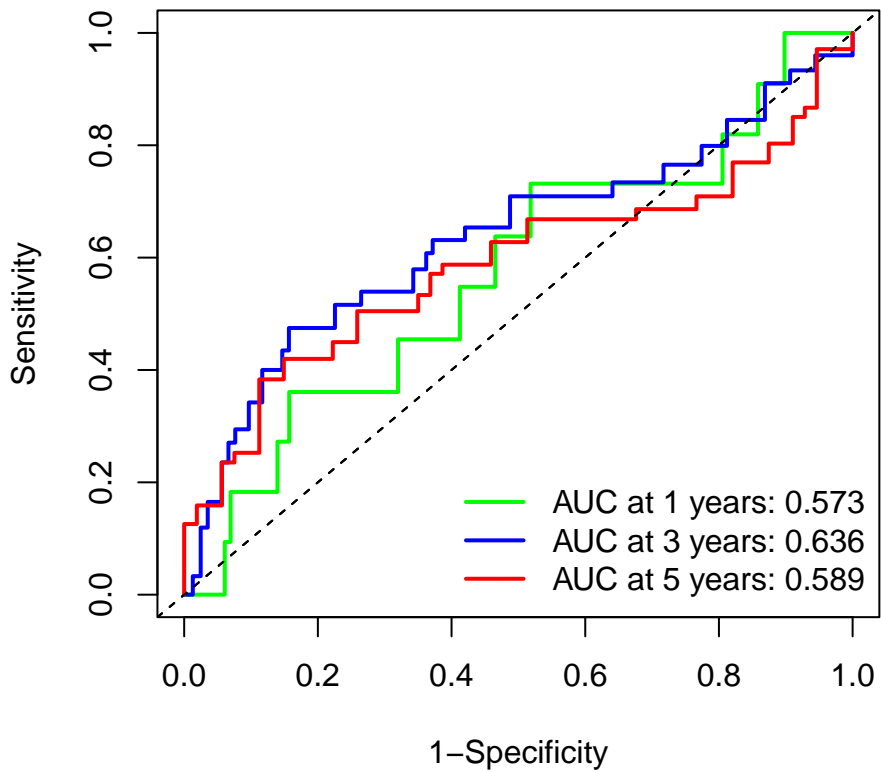

Supplement: Supplementary file 6 — Supplementary Figure S2. [file 41598_2022_22879_MOESM6_ESM.pdf]

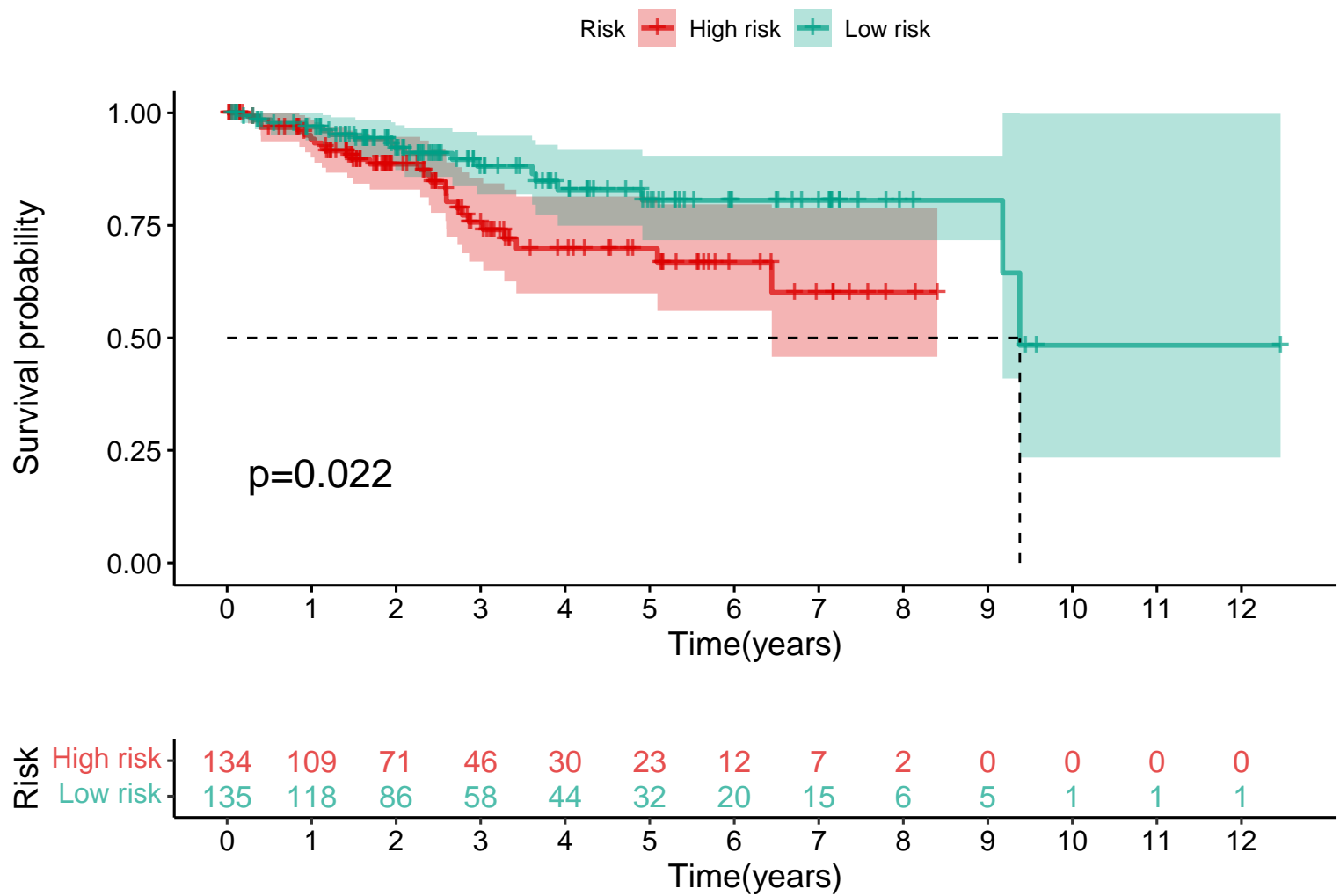

Supplement: Supplementary file 7 — Supplementary Figure S2. [file 41598_2022_22879_MOESM7_ESM.pdf]
